# Everolimus-Involving Immunosuppression Attenuates Renal Interstitial Fibrosis Through Modulation of Mammalian Target of Rapamycin-Related Signal Transduction

**DOI:** 10.3390/ijms27177634

**Published:** 2026-08-26

**Authors:** Takuro Saito, Mitsuru Saito, Ryohei Yamamoto, Ryuichiro Sagehashi, Yu Aoyama, Mizuki Mori, Chika Kajiwara, Kengo Furihata, Hideaki Kagaya, Hironobu Yagishita, Nobuhiro Fujiyama, Soki Kashima, Kazuyuki Numakura, Shintaro Narita, Masafumi Kikuchi, Masatomo Miura, Tomonori Habuchi

**Affiliations:** 1 Department of Urology, Graduate School of Medicine, Akita University, 1-1-1 Hondo, Akita 010-8543, Japan; tkr.526.lab@gmail.com (T.S.); sagehashi1001@gmail.com (R.S.);; 2 Division of Blood Purification, Akita University Hospital, 1-1-1 Hondo, Akita 010-8543, Japan; 3 Center for Kidney Disease and Transplantation, Akita University Hospital, 1-1-1 Hondo, Akita 010-8543, Japan; 4 Department of Pharmacy, Akita University Hospital, 1-1-1 Hondo, Akita 010-8543, Japanmasafumi.kikuchi@hos.akita-u.ac.jp (M.K.);; 5 Clinical Research Promotion and Support Office, Future Cooperative Research Organization, Akita University, 1-1-1 Hondo, Akita 010-8543, Japan; 6 Department of Renal and Urologic Surgery, Asahikawa Medical University, 2-1-1-1 Midorigaoka Higashi, Asahikawa 078-8510, Japan; 7 Department of Pharmacokinetics, Graduate School of Medicine, Akita University, 1-1-1 Hondo, Akita 010-8543, Japan

**Keywords:** mammalian target of rapamycin inhibitor, mammalian target of rapamycin-related protein, interstitial fibrosis, living kidney transplantation, kidney transplant outcomes

## Abstract

Chronic allograft dysfunction, driven by interstitial fibrosis and tubular atrophy, remains a major cause of long-term renal allograft loss. Everolimus (EVR), a mammalian target of rapamycin (mTOR) inhibitor, may reduce fibrosis through antifibrotic effects and calcineurin inhibitor minimization. This study aimed to evaluate the impact of EVR-involving immunosuppression on renal allograft fibrosis and to identify predictors of fibrotic progression. A total of 104 living-donor kidney transplant recipients transplanted between 2011 and 2017 were retrospectively analyzed (EVR, *n* = 61; non-EVR, *n* = 43). Interstitial fibrosis was quantified in protocol biopsies using digital image analysis. Phosphorylation of the mTOR-signaling proteins p70 ribosomal S6 kinase and eukaryotic translation initiation factor 4E-binding protein 1 was assessed using a semiquantitative immunoreactive score. Multivariable regression analyses identified independent predictors of fibrotic progression. Cytomegalovirus infection was less frequent in the EVR group, whereas acute rejection, graft function, and graft survival were comparable between groups. At 1-year posttransplantation, interstitial fibrosis was significantly lower in the EVR group. EVR significantly suppressed p-4EBP1 phosphorylation and effectively modulated the mTOR-signaling pathway. Multivariable analysis identified the absence of EVR therapy as an independent predictor of accelerated fibrosis. EVR-involving immunosuppression attenuated renal interstitial fibrosis, likely through suppression of mTOR-signaling.

## 1. Introduction

Since the early 2000s, short-term outcomes after kidney transplantation (KTx) have improved substantially, largely owing to the introduction of calcineurin inhibitor (CNI)-based immunosuppressive regimens, particularly those incorporating tacrolimus (TAC) [1]. Despite these advances, CNI-induced nephrotoxicity remains a major obstacle to long-term graft survival, as it promotes interstitial fibrosis and tubular atrophy, one of the principal causes of chronic allograft dysfunction and loss [2,3]. Beyond causing direct tubular injury, CNIs contribute to renal fibrosis by stimulating the secretion of vasoconstrictors, including thromboxane and endothelin, in vascular smooth muscle and endothelial cells, which triggers afferent arteriolar vasoconstriction and subsequent hypoperfusion, thereby driving the fibrotic process [4].

In recent years, several strategies have been investigated to minimize CNI-induced nephrotoxicity, including the co-administration of mammalian target of rapamycin inhibitors (mTORis) with low-dose CNIs [5,6,7,8] and the complete conversion from CNI-based to mTORi-based immunosuppressive regimens [9,10]. In addition to their immunosuppressive effects, mTORis have demonstrated antifibrotic effects in the kidneys across various preclinical models [11,12] and clinical cohorts [5,6,7,13]. These findings suggest that mTORis may serve as pivotal therapeutic agents for improving long-term allograft outcomes by exerting both direct inhibitory effects on fibrogenesis and indirect benefits by facilitating reduced CNI exposure.

The primary objective of this study was to evaluate the effect of everolimus (EVR)-based immunosuppressive regimens on cortical interstitial fibrosis and to identify independent predictors of fibrotic progression. While previous studies have suggested that EVR-based regimens may attenuate fibrosis in the renal graft by downregulating mTOR-signaling pathways [5,7] without compromising safety or efficacy [6], several important questions remain unresolved. In particular, the relative contribution of EVR therapy compared with other clinical determinants—and more crucially, its correlation with the detailed pharmacokinetics of immunosuppressants like TAC—has not been fully elucidated.

The present study aimed to compare clinical outcomes between patients receiving a conventional immunosuppressive regimen (non-EVR group) and those receiving an EVR-based regimen (EVR group). Digital image analysis was used for the longitudinal and quantitative evaluation of the interstitial fibrosis rate (IFR) within the renal cortex of the renal allograft. Using 0 h biopsy specimens as a baseline, the temporal progression of IFR was calculated for comparative analysis. Furthermore, the impact of EVR on the phosphorylation of key mTOR-signaling proteins, including p70 ribosomal S6 kinase (P70S6K) and eukaryotic initiation factor 4E-binding protein-1 (4EBP1), was assessed using immunohistochemical scoring. Finally, multivariable analyses incorporating detailed pharmacokinetic parameters of TAC and other clinical variables were performed to identify independent predictors of accelerated interstitial fibrosis.

## 2. Results

### 2.1. Baseline Characteristics and Clinical Outcomes

The baseline characteristics of the EVR and non-EVR groups are summarized in Table 1. In accordance with the chronological study design, the mean observation period was significantly longer in the non-EVR group. Patients in the EVR group were significantly older at the time of KTx and showed a higher prevalence of preemptive transplantation, CYP3A5*1 allele carriers, and successful PSL withdrawal. Regarding primary kidney diseases, chronic glomerulonephritis was significantly less frequent, whereas nephrosclerosis was more prevalent in the EVR group. Regarding donor characteristics, the EVR group included donors with a significantly lower preoperative estimated glomerular filtration rate. This factor may have contributed to the significantly higher baseline IFR observed in the 0 h biopsy specimens of this group.

**Figure 1:**
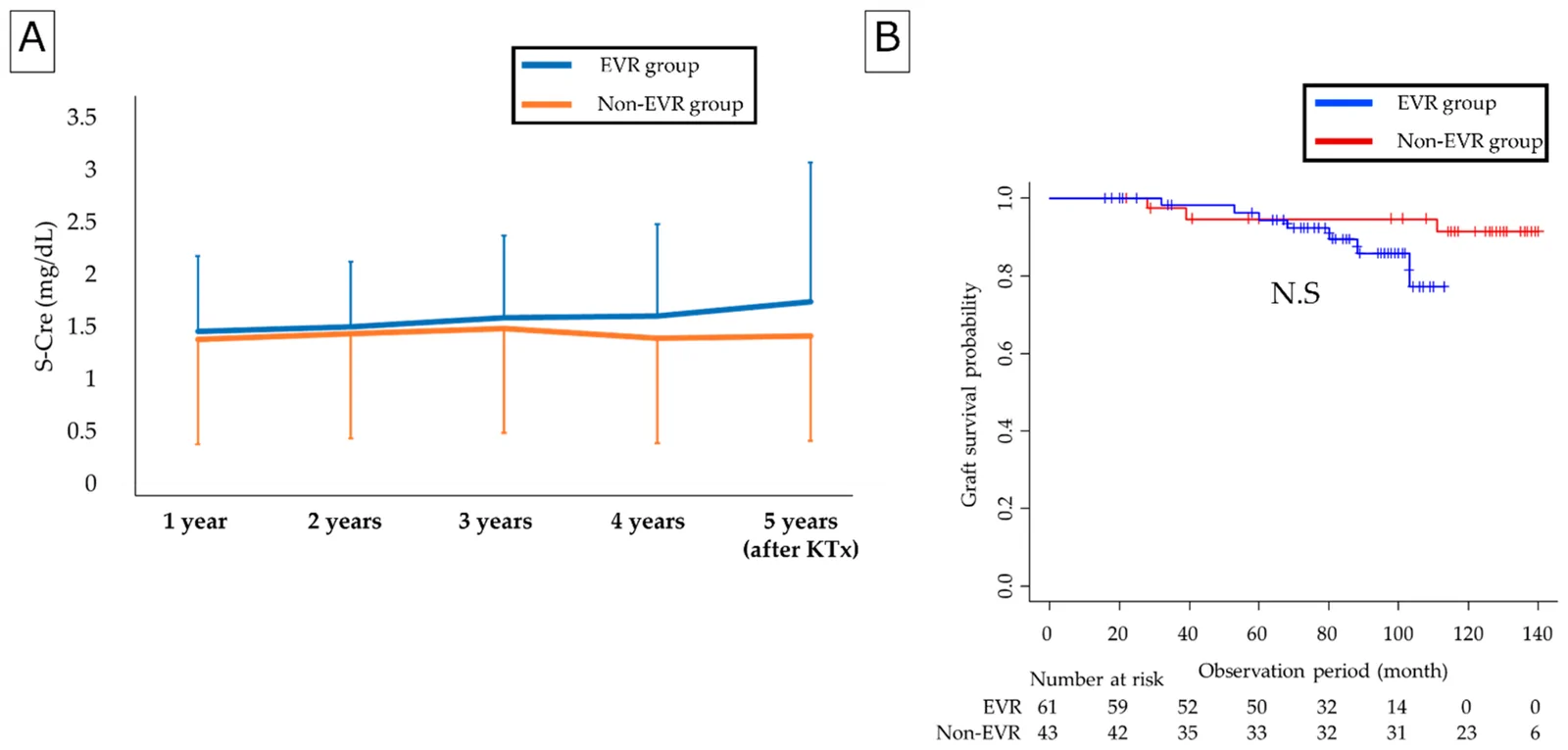
Comparison of clinical outcomes between the everolimus (EVR) and non-EVR groups. (**A**) Longitudinal changes in serum creatinine levels during the 5-year follow-up period after kidney transplantation (KTx). Data are presented as mean ± standard deviation. No significant differences were observed between the EVR and non-EVR groups at any time point. (**B**) Kaplan–Meier analysis of overall graft survival. No significant difference in graft survival was observed between the two groups (HR = 3.12, 95% CI: 0.66–14.64, *p* = 0.149). KTx, kidney transplantation; N.S., not significant.

A comparison of clinical outcomes and immunosuppressive pharmacokinetics is presented in Table 2 and Figure 1. Consistent with previous reports, the incidence of cytomegalovirus (CMV) infection (defined as antigenemia > 1 positive cell on two slides via the C10/C11 assay) was significantly lower in the EVR group. Regarding CMV risk profile, the distribution of donor/recipient (D/R) CMV serostatus showed no significant difference between the groups (D+/R− status: 9.3% in the non-EVR group vs. 16.4% in the EVR group, *p* = 0.126), and all D+/R− status patients were managed under a unified institutional CMV prophylaxis protocol. Importantly, this reduction did not compromise clinical efficacy, as the rates of acute rejection (including subclinical rejection) and corticosteroid pulse therapy were comparable between the EVR and the non-EVR groups (Table 2). The rate of de novo donor-specific antibody production at 1-year posttransplantation was also comparable between the groups (with one case observed in each group). Furthermore, no significant differences were observed between the two groups in graft function (Figure 1A) or overall graft survival during the 5-year follow-up period (Figure 1B; HR = 3.12, 95% CI: 0.66–14.64, *p* = 0.149). Regarding pharmacokinetic parameters, the EVR group exhibited significantly lower mycophenolic acid (MPA) area under the concentration–time curve from 0 to 12 h (AUC_0–12_) at 1-month posttransplantation, as well as significantly lower TAC AUC_0–24_ and MPA AUC_0–12_ values at one year after KTx (Table 2).

### 2.2. Longitudinal Changes in IFR

**Figure 2:**
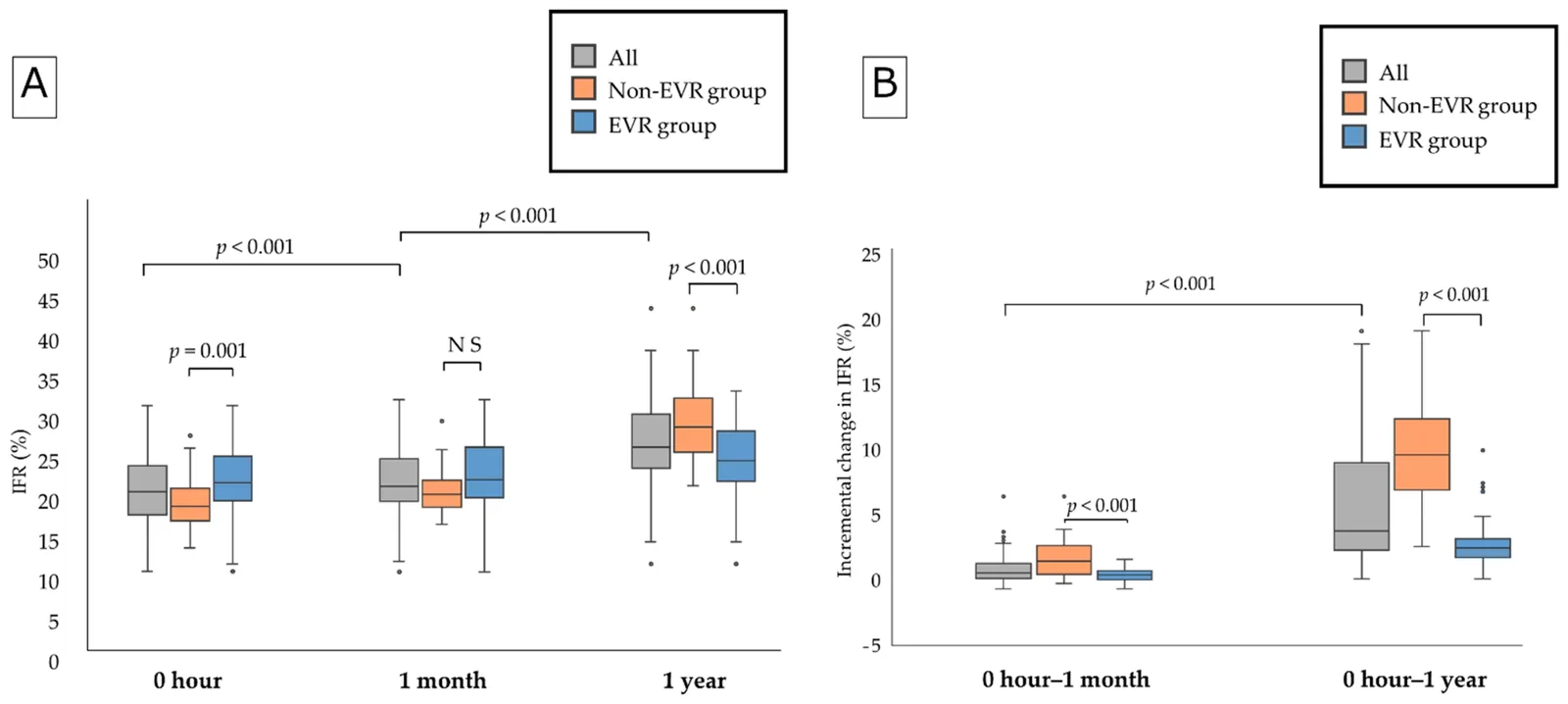
Quantitative evaluation of interstitial fibrosis rate (IFR) using digital image analysis. (**A**) Longitudinal changes in the interstitial fibrosis rate (IFR) within the renal cortex. Note that although the everolimus (EVR) group exhibited a significantly higher baseline IFR (in the 0 h biopsy specimen), this trend was reversed 1 year after transplantation, with the non-EVR group demonstrating a significantly higher fibrotic area (*p* < 0.001). (**B**) Comparison of changes in fibrosis formation from baseline. The increase in IFR was significantly attenuated in the EVR group compared with the non-EVR group at both one month and one year after kidney transplantation (*p* < 0.001 for both). Data are presented as box-and-whisker plots showing the median, interquartile range, and range. Outliers are indicated by individual dots. IFR, interstitial fibrosis rate; EVR, everolimus; N.S., not significant.

The IFR increased significantly over time in the overall cohort (Figure 2A). At baseline (0 h biopsy), the EVR group exhibited a significantly higher IFR than the non-EVR group; however, this difference was no longer evident at 1-month posttransplantation, and the relationship was reversed by 1 year, with the non-EVR group demonstrating significantly greater fibrotic formation (Figure 2A). The progression of fibrosis relative to the baseline (0 h) biopsy is presented in Figure 2B. While the increase in IFR was significantly greater in the non-EVR group as early as one month after KTx, this disparity became substantially more pronounced by 1-year posttransplantation (Figure 2B). Furthermore, to adjust for potential baseline imbalances and the effect of regression to the mean, analysis of covariance (ANCOVA) was performed using the 1-year IFR as the outcome variable and the baseline (0 h) IFR as a covariate. The analysis confirmed that the 1-year IFR remained significantly lower in the EVR group compared with the non-EVR group (adjusted coefficient = −6.78%, *p* < 0.001), demonstrating that the antifibrotic effect of EVR was independent of initial baseline fibrosis levels.

### 2.3. Longitudinal Changes in the Immunoreactive Score (IRS) of Phosphorylated mTOR-Related Proteins

**Figure 3:**
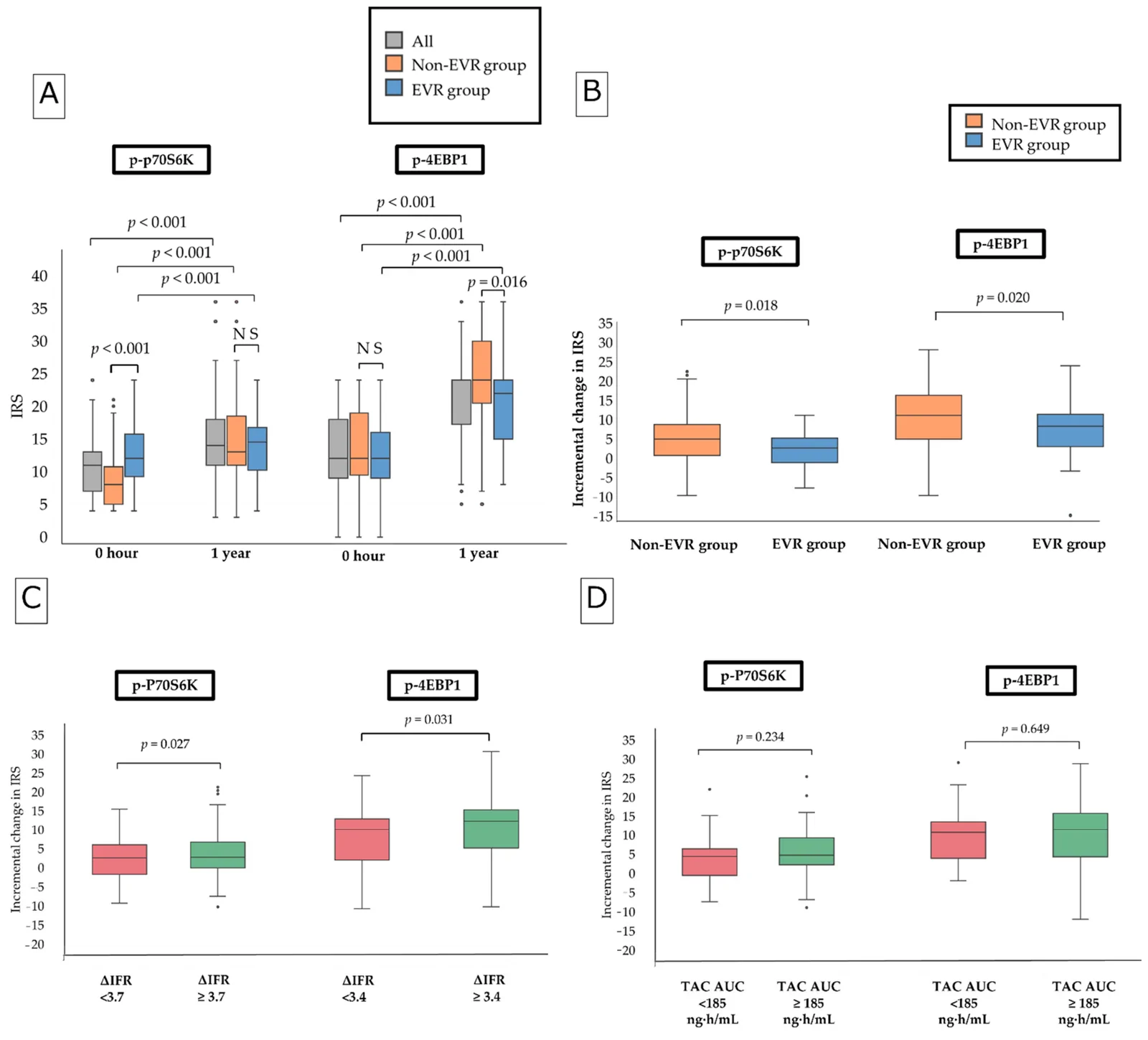
Longitudinal changes in the expression of phosphorylated mammalian target of rapamycin (mTOR)-related proteins. (**A**) Comparison of the immunoreactive score (IRS) for phosphorylated p70 ribosomal S6 kinase (p-p70S6K) and phosphorylated eukaryotic translation initiation factor 4E-binding protein 1 (p-4EBP1) between the groups. Both groups showed significant increases in protein phosphorylation at 1 year compared to baseline (*p* < 0.001). Notably, the everolimus (EVR) group exhibited a significantly lower p-4EBP1 IRS at 1-year posttransplantation. (**B**) Comparison of the incremental change in IRS from baseline to 1 year. The EVR group demonstrated significantly smaller increases in the phosphorylation of both p-p70S6K and p-4EBP1, indicating a potent inhibitory effect on mTOR-signaling activity. Data are presented as box-and-whisker plots. (**C**) Comparison of the incremental change in IRS according to the degree of fibrosis progression. Patients were stratified into two groups based on the median increase in interstitial fibrosis rate over 1 year (3.7% for p-p70S6K analysis and 3.4% for p-4EBP1 analysis). High fibrosis progressors exhibited significantly greater increases in protein phosphorylation than those with less fibrotic progression. (**D**) Comparison of the incremental change in IRS according to tacrolimus (TAC) exposure. Patients were stratified according to the median TAC area under the concentration–time curve (AUC) at 1 year after kidney transplantation (185 ng·h/mL). No significant correlation was observed between TAC exposure and the degree of mTOR-related protein phosphorylation. Data are presented as box-and-whisker plots. EVR, everolimus; IRS, immunoreactive score; IFR, interstitial fibrosis rate; TAC, tacrolimus; AUC, area under the concentration curve.

Across the entire cohort as well as within each treatment group, the IRSs for p-P70S6K and p-4EBP1 increased significantly from baseline to 1 year after KTx (Figure 3A). With respect to p-P70S6K expression, the EVR group exhibited a significantly higher IRS at baseline than the non-EVR group; however, this between-group difference was no longer evident at 1-year posttransplantation (Figure 3A). In contrast, no significant difference was observed in the p-4EBP1 IRS at baseline, and the EVR group demonstrated a significantly lower IRS than the non-EVR group 1 year after KTx (Figure 3A).

### 2.4. Increments in the IRS and Their Associations

The increments in IRS from baseline to 1-year posttransplantation for both p-P70S6K and p-4EBP1 were significantly smaller in the EVR group than in the non-EVR group (Figure 3B). The entire cohort was stratified according to the median increase in IFR at 1 year. Patients with higher IFR increments exhibited significantly higher IRS values for both p-P70S6K and p-4EBP1 (Figure 3C). In contrast, no significant differences in the IRS of either protein were observed when the cohort was stratified according to the median TAC AUC from 0 to 24 h, suggesting that mTOR-signaling activity was not significantly influenced by differences in TAC exposure in this cohort (Figure 3D).

### 2.5. Risk Factors for IFR Progression

Univariable analysis identified several risk factors significantly associated with IFR progression: these included the absence of rituximab induction therapy, CYP3A5*3/*3 status, hypertensive nephrosclerosis as the primary kidney disease, omission of EVR from the immunosuppressive regimen, MPA AUC_0–12_ ≥ 40 μg·h/mL at one month, TAC AUC_0–24_ ≥ 185 ng·h/mL at 1 year, and a baseline IFR ≥ 21.2% in the 0 h biopsy specimen (Table 3). Notably, in the multivariable analysis, EVR administration was the only variable that remained significantly associated with IFR progression (*p* < 0.001). This indicates that EVR administration is an independent protective factor against the progression of interstitial fibrosis in the renal allograft (Table 3).

## 3. Discussion

In the present study, we demonstrated that an immunosuppressive regimen based on EVR significantly attenuated renal interstitial fibrosis at 1-year post-KTx compared to a conventional EVR-free regimen. This antifibrotic effect was closely associated with the suppression of mTOR-related protein phosphorylation, specifically p70S6K and 4EBP1. Multivariable analysis demonstrated EVR-involving regimens to be an independent protective factor against fibrotic progression. In addition, the clinical profile of the EVR-involving regimen was favorable; despite a significantly lower incidence of CMV infection, the rates of acute rejection remained comparable between the two groups.

Intracellularly, mTOR exists as two distinct multiprotein complexes: mTOR complex 1 (mTORC1) and mTOR complex 2 (mTORC2). mTOR inhibitors, including EVR, primarily target mTORC1, thereby modulating critical cellular processes—such as growth, proliferation, metabolism, and angiogenesis—through inhibition of downstream targets such as p70S6K and 4EBP1 within the transforming growth factor-beta (TGF-β) signaling pathway [14]. Accumulating evidence has highlighted a pivotal role for mTOR signaling in the pathogenesis of renal fibrosis in chronic kidney disease [11,15,16]. Consistent with these findings, several studies have demonstrated that increased expression of TGF-β and mTOR-related proteins is associated with the progression of interstitial fibrosis and tubular atrophy post-KTx [5,7,13]. Becker et al. reported that conversion from CNIs to mTORis was associated with reduced tubular expression of TGF-β and interstitial fibrosis, whereas continued CNI therapy was not [13]. Similarly, Nishioka et al. [5] investigated mTOR-related protein expression using an immunosuppressive protocol comparable to ours (low-dose TAC plus EVR) and demonstrated significantly lower expression of p-4EBP1 in the EVR group, consistent with our findings. However, some reports suggest that the behavior of mTOR-related proteins may be heterogeneous; for instance, in TGF-β-stimulated fibroblasts, p70S6K expression was shown to correlate with mTOR levels, while 4EBP1 expression did not [17]. Furthermore, a study on sirolimus conversion [18] reported no significant reduction in fibrotic area despite preserved graft function, suggesting that antifibrotic efficacy may vary among different mTOR inhibitors.

As shown in Figure 3A, the baseline (0 h) IRS for p-P70S6K was significantly higher in the EVR group (*p* < 0.001). Consequently, evaluating absolute values at 1 year alone does not fully reflect the dynamic treatment effect. When evaluating the change from baseline to 1 year (ΔIRS, Figure 3B), the increase in p-P70S6K IRS was significantly suppressed in the EVR group compared to the non-EVR group (*p* = 0.018). This demonstrates that EVR effectively attenuated the progressive hyperphosphorylation of P70S6K over time.

In addition, the difference in phosphorylation dynamics between p-P70S6K and p-4EBP1 can be explained by the well-established S6K1/IRS-1/PI3K negative feedback loop [19,20]. Chronic inhibition of mTORC1 releases the S6K1-mediated negative feedback on IRS-1, leading to compensatory upstream PI3K/Akt reactivation, which partially blunts the sustained suppression of P70S6K phosphorylation over time [21]. In contrast, 4EBP1 phosphorylation is independent of this S6K1-mediated feedback loop, making p-4EBP1 a more direct, robust, and sensitive biomarker for long-term intrarenal mTORC1 inhibition. A similar phenomenon was reported in a clinical KTx cohort by Nishioka et al. [5].

A distinguishing feature of our study is the integration of detailed TAC and MPA pharmacokinetic data with the quantitative assessment of interstitial fibrosis. Given that CNI nephrotoxicity is a major contributor to fibrotic progression in renal allografts [2,3,4], evaluating its correlation with TAC exposure is essential. We previously demonstrated that in an era of higher target TAC trough levels, patients with the CYP3A5*3/*3 genotype exhibited significantly higher TAC trough levels, greater interstitial fibrosis, and a higher incidence of chronic allograft nephropathy compared with those with the CYP3A5*1 allele [22,23]. Subsequently, by adopting a genotype-based protocol—incorporating a lower initial dose and reduced target trough concentrations for patients with the CYP3A5*3/*3 genotype—the disparity in fibrotic progression between genotypes was abolished [23]. In the present study, while the lower target TAC trough levels used in the EVR group likely contributed to the mitigation of fibrosis, multivariable analysis revealed that high TAC AUC at one month and one year was not an independent predictor of fibrotic progression. Given that patients with the CYP3A5*3/*3 genotype in this cohort were managed using a genotype-guided dosing protocol, the impact of *CYP3A5* polymorphism on fibrosis may have been further minimized by the lower overall TAC exposure. Crucially, the significantly higher expression of phosphorylated mTOR-related proteins observed in patients with accelerated fibrotic progression suggests that mTOR signaling remains a more potent determinant of fibrogenesis than the CNI exposure levels in modern protocols.

In selecting CNI-sparing strategies, alternative agents such as belatacept have shown promising long-term allograft outcomes in global clinical trials; however, belatacept remains unapproved under national health insurance in Japan. Therefore, an EVR-involving protocol combined with reduced-dose CNI represents the most practical and validated strategy in routine Japanese clinical practice. Beyond facilitating CNI dose minimization, EVR provides multifaceted synergistic benefits, including direct antifibrotic action via mTORC1 inhibition, anti-oncogenic activity, and antiviral protection against CMV.

The relationship between clinical variables and fibrotic progression also warrants consideration. The protective effect of EVR against CMV infection has been well-documented [24,25], and our findings are corroborated by our data. Given the comparable CMV D/R serostatus distribution between groups and the application of a uniform prophylaxis protocol, the lower incidence of CMV infection in the EVR group likely reflects the added protective effect of EVR, consistent with its established antiviral properties. Although CMV infection has been implicated in the exacerbation of renal fibrosis [26], it was not an independent risk factor in our multivariable analysis. Regarding corticosteroids, while some studies have suggested antifibrotic potential [27], more recent evidence indicates that their impact on fibrosis may be limited [28]. Similarly, our results showed no correlation between fibrotic progression and corticosteroid withdrawal or a history of steroid pulse therapy.

Despite evidence suggesting that fibrotic progression at 1 year is associated with poorer long-term graft outcomes [3], we did not observe significant superiority in graft function or survival in the EVR group compared to the non-EVR group. Several factors may have contributed to this finding, including the relatively small sample size and the significantly lower baseline donor graft function in the EVR group. Furthermore, our previous study using an independent cohort similarly demonstrated no difference in graft survival even when comparing the most fibrotic cases (top 20 cases) with the rest of the cohort [29]. Although inhibiting fibrosis is theoretically advantageous, fibrogenesis in the renal allograft may become a more decisive prognostic factor when accompanied by specific inflammatory cell infiltration [30,31].

This study has several limitations. First, as a single-center historical cohort study, the sample size was insufficient to perform propensity score matching, and potential era-dependent confounders cannot be completely ruled out, although multivariable regression and ANCOVA were performed to adjust for baseline imbalances. Second, although p-P70S6K and p-4EBP1 were evaluated as the principal downstream effectors of mTORC1, other signaling components (such as mTORC2-related molecules or upstream regulators) were not comprehensively assessed, partly due to the limited availability of archived tissue specimens. Third, there were inherent differences in patient backgrounds and observation periods between the groups. Fourth, we did not evaluate the impact of varying EVR trough levels. Although the target EVR trough level in our protocol was 3–5 ng/mL, the clinically accepted upper limit is 8 ng/mL. Therefore, whether higher EVR exposure could further suppress mTOR phosphorylation and further enhance the antifibrotic effects warrants further investigation.

## 4. Materials and Methods

### 4.1. Patient Population and Study Design

**Figure 4:**
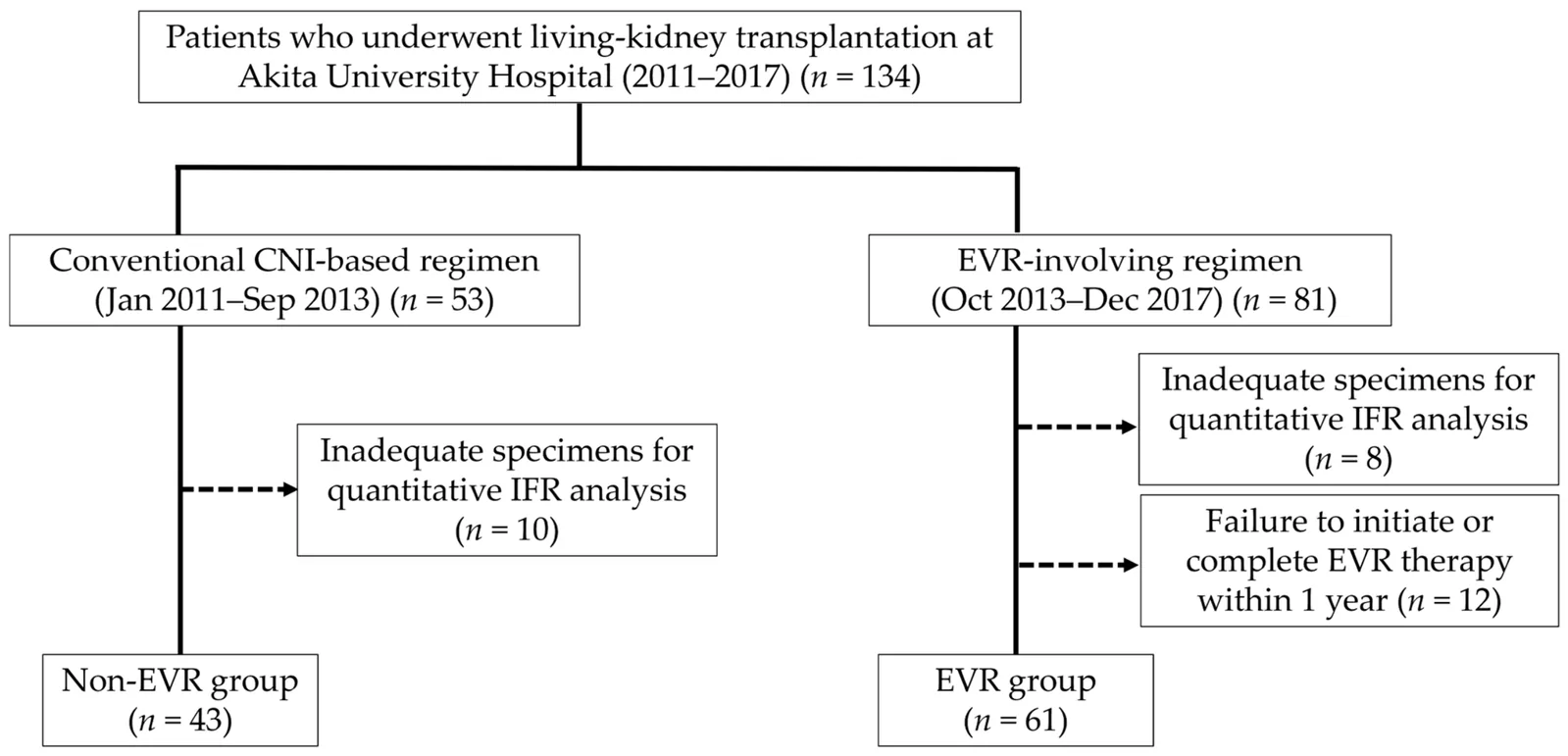
Flow diagram of the patient selection process. Flowchart illustrating the patient selection process. A total of 134 recipients of living-donor kidney transplantation were initially screened. Patients were stratified into two chronological cohorts: the non-everolimus (non-EVR) group (January 2011–September 2013) and the EVR group (October 2013–December 2017). After excluding patients with biopsy specimens unsuitable for quantitative digital image analysis and those who discontinued EVR within 1 year after transplantation, 43 patients in the non-EVR group and 61 patients in the EVR group were included in the final analysis.

A total of 134 consecutive patients who underwent living-donor KTx at Akita University Hospital between 2011 and 2017 were initially included in this study. Patients were stratified into two groups based on their immunosuppressive regimen: the non-EVR group (*n* = 53), comprising those who received a conventional immunosuppressive regimen prior to September 2013, and the EVR group (*n* = 81), comprising those who received an EVR-based regimen from October 2013 onward. To ensure the validity of the longitudinal analysis, the following exclusion criteria were applied: (i) biopsy specimens were unsuitable for quantitative image analysis, precluding accurate temporal IFR measurement, and (ii) failure to initiate or complete EVR therapy for at least one year (*n* = 12). Reasons for non-initiation or discontinuation included non-initiation prior to EVR administration due to stomatitis (*n* = 1) or lymphocele (*n* = 2); premature discontinuation following EVR initiation due to drug eruption (*n* = 1), hyperglycemia (*n* = 1), stomatitis (*n* = 1), or chylous ascites (*n* = 1); and discontinuation for unknown reasons, including patient refusal (*n* = 5). Consequently, 43 patients in the non-EVR group and 61 patients in the EVR group were included in the final analysis (Figure 4).

### 4.2. Immunosuppressive Protocols

The non-EVR group received a conventional triple-drug immunosuppressive regimen consisting of TAC, mycophenolate mofetil (MMF), and prednisolone (PSL). In accordance with our previously established protocols [22,23,32], the initial TAC dose was individualized based on *CYP3A5* genotype: patients with the enzyme-deficient CYP3A5 *3/*3 genotype received 0.2 mg/kg/day, whereas carriers of the CYP3A5 *1 allele received 0.3 mg/kg/day. TAC administration was initiated 2 days before KTx, and target trough concentrations were maintained at 10–12 ng/mL until postoperative week (POW) 1, 8–10 ng/mL during POW 1–2, 6–8 ng/mL during POW 2–4, and 5–7 ng/mL thereafter. MMF was initiated at 1500 mg/day 2 days before transplantation and maintained unless adverse events occurred. PSL was initiated at 500 mg on the day of transplantation, tapered intravenously to 40 mg/day by POW 1, converted to oral administration and tapered to 10 mg/day by POW 2, and further reduced to 5 mg/day six months after KTx. In patients with underlying glomerulonephritis or autoimmune diseases, PSL was maintained at a minimum dose of 5 mg/day, and withdrawal was pursued whenever feasible in patients with diabetes mellitus, nephrosclerosis, or autosomal dominant polycystic kidney disease.

In the EVR group, EVR therapy was initiated at POW 2 with a target trough level of 3–5 ng/mL, concomitant with an MMF dose reduction from 1500 to 1000 mg/day. The TAC dosing protocol remained identical to that used in the non-EVR group until POW 4, after which the target trough level was reduced to 4–5 ng/mL. The dose of the PSL tapering regimen was identical to that used in the non-EVR group.

All patients received 20 mg of basiliximab intravenously on the day of transplantation and again on postoperative Day 4 as induction therapy. In immunologically high-risk recipients, including those undergoing ABO-blood-group-incompatible transplantation or those who were donor-specific human leukocyte antigen–antibody-positive, desensitization was performed using a single dose of rituximab (200 mg/body), with additional antibody removal procedures as clinically indicated. Acute T-cell-mediated rejection was treated with high-dose PSL pulse therapy, whereas antibody-mediated rejection was managed with a combination of antibody removal therapies, PSL pulse therapy, and/or intravenous immunoglobulin.

### 4.3. Quantitative Histological Image Analysis of Protocol Biopsies

Protocol renal allograft biopsies were performed at 0–1 h posttransplantation (baseline) and at 1, 6, and 12 months after KTx. Formalin-fixed, paraffin-embedded renal tissue samples were sectioned at a thickness of 2 μm and stained using hematoxylin and eosin, periodic acid–Schiff, Elastica–Masson (EM), and periodic acid-methenamine silver stains. For the present study, pre-implantation biopsy specimens obtained during bench surgery at baseline (0 h), as well as specimens obtained at one-month and one-year post-KTx, were utilized for analysis.

EM-stained glass slides were digitized using a virtual slide scanner (NanoZoomer 2.0-RS C10730-13; Hamamatsu Photonics Co., Shizuoka, Japan). Digital images were acquired at ×100 magnification using NanoZoomer Digital Pathology View 2 software and subsequently imported into dedicated image analysis software (WinROOF 2015 version 3.7.0; Mitani Co., Fukui, Japan) for further processing. Within the software, the renal cortical regions were manually delineated. In accordance with our previously established methodology [32], glomeruli, blood vessels, medulla and corticomedullary junction areas, and subcapsular fibrotic regions were excluded from the analysis.

Fibrotic regions, characterized by light-blue staining in the EM-stained sections, were identified using a standardized color-thresholding technique. Specifically, the RGB (red, green, and blue) color space was calibrated for each specimen to ensure accurate detection of all light-blue-stained areas, which were subsequently classified as positive signals (green regions within the software interface) [32]. The IFR was calculated as the ratio of the total fibrotic area to the total cortical area. Furthermore, the progression of fibrosis was assessed by quantifying longitudinal changes in IFR at 1-month and 1-year posttransplantation relative to the baseline (0 h biopsy) IFR. To minimize potential observer bias, image quantification was performed in a blinded manner. Specifically, all specimens were analyzed sequentially by time point (first all 0 h specimens, followed by 1-month and 1-year specimens), with patient identification, clinical outcomes, and group assignments (EVR vs. non-EVR) blinded. This time-point-based analysis workflow inherently concealed individual longitudinal clinical courses during quantification. Furthermore, because the analysis relied on a standardized, automated digital RGB color-thresholding algorithm using WinROOF software rather than visual semi-quantitative scoring, subjective criteria and intraobserver variability were inherently minimized. This image processing workflow was established based on previously reported protocols from our institution [23,32].

### 4.4. Pharmacokinetic Assessment of Immunosuppressants

To characterize the pharmacokinetic profiles of TAC, MMF, and EVR, blood samples were collected at nine distinct time points—immediately prior to administration (trough, 0 h) at 9:00 AM and at 1, 2, 3, 4, 6, 9, 12, and 24 h postadministration. Sampling was performed at both 1-month and 1-year posttransplantation. The area under the blood (plasma) concentration–time curve (AUC) was then calculated from these serial measurements.

Blood concentrations of each immunosuppressive agent were determined using standardized assays: TAC levels were measured by chemiluminescence immunoassay, while the plasma concentration of MPA, the active metabolite of MMF, was quantified using high-performance liquid chromatography, and EVR concentrations were assessed using electrochemiluminescence immunoassay [33].

### 4.5. Immunohistochemical Analysis of Phosphorylated mTOR-Related Proteins

To assess activation of the mTOR-signaling pathway, immunohistochemical staining for downstream effector proteins, specifically phosphorylated P70S6K (p-P70S6K) and phosphorylated 4EBP1 (p-4EBP1), was performed on biopsy specimens obtained at baseline (0 h) and 1-year post-KTx. These two phosphorylated proteins were selected as they represent the canonical and direct downstream effectors of mTORC1 signaling, serving as established key markers for evaluating intrarenal mTOR pathway activation in kidney transplantation, as demonstrated in previous clinical studies [5,7].

Formalin-fixed, paraffin-embedded tissue sections were cut at a thickness of 2 μm. Following deparaffinization and rehydration with distilled water, heat-induced antigen retrieval was performed using citrate buffer (pH 6.0) in an autoclave at 105 °C for 10 min, followed by gradual cooling to room temperature. Endogenous peroxidase activity was quenched by incubating the sections with 0.3% hydrogen peroxide in methanol for 30 min. Nonspecific binding was subsequently blocked by incubation with 10% normal goat serum for 30 min.

The sections were then incubated overnight at 4 °C with the following primary antibodies: anti-p-P70S6Kα (A-6; Santa Cruz Biotechnology, Inc., Dallas, TX, USA) at a 1:400 dilution or anti-p-4EBP1 (Thr37/46; Cell Signaling Technology, Inc., Danvers, MA, USA) at a 1:200 dilution. After three washes with Tris-buffered saline, the sections were incubated for 30 min with horseradish peroxidase-conjugated secondary antibodies (anti-rabbit IgG, DAKO 4003; or anti-mouse IgG, DAKO 4001; Agilent Technologies, Inc., Santa Clara, CA, USA). Immunoreactivity was visualized using a 3,3′-diaminobenzidine tetrahydrochloride solution. Finally, the sections were counterstained with Mayer’s hematoxylin, dehydrated, cleared, and mounted. Prior to the main study, optimal primary antibody concentrations were predetermined through serial titration tests (1:50 to 1:400) using positive control tissues (human colon adenocarcinoma for p-4EBP1 and normal esophageal mucosa for p-P70S6K). Negative controls omitting the primary antibodies were routinely run in parallel to confirm the absence of non-specific background staining. Specific immunoreactivity in kidney allograft specimens was confirmed predominantly in the cytoplasm and partially in the nuclei of proximal and distal renal tubular epithelial cells.

### 4.6. Assessment of mTOR-Related Protein Expression via Immunoreactive Score

**Figure 5:**
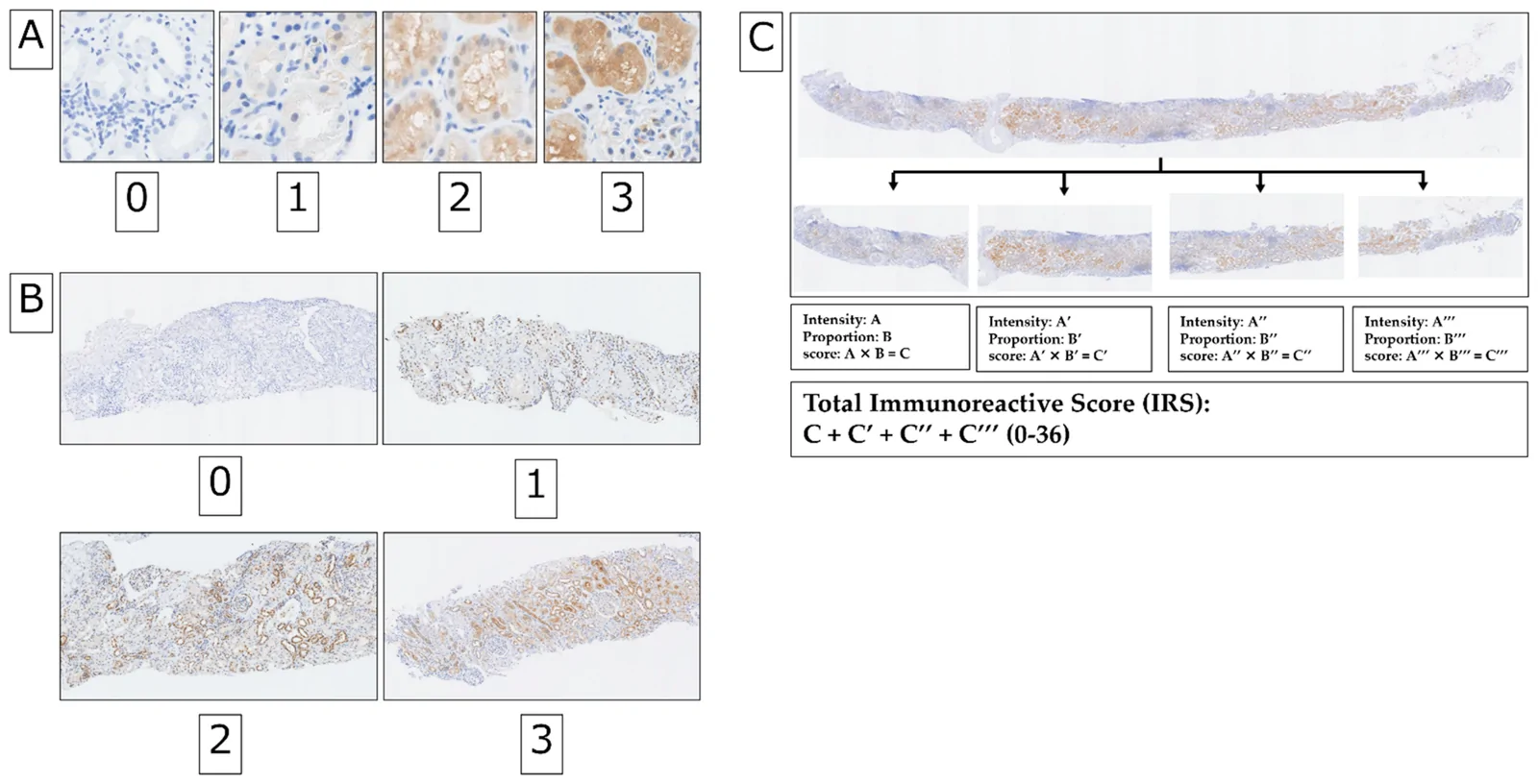
Representative images and calculation of the immunoreactive score (IRS). (**A**) Semiquantitative assessment of staining intensity in tubular epithelial cells, graded on a scale of 0–3 (0: negative, 1: weak, 2: moderate, 3: strong). (**B**) Assessment of the proportion of positively stained area, graded on a scale of 0–3. (**C**) Calculation of the total IRS. To account for intraparenchymal heterogeneity in staining patterns, each biopsy specimen was divided into four quadrants. The IRS for each quadrant was calculated as the product of the staining intensity score and the positive staining area score (IRS = intensity score x area score). The final IRS was defined as the sum of the scores from all four quadrants (total range: 0–36). Original magnification: ×20.

The expression intensity of phosphorylated mTOR-related proteins was evaluated using the IRS, according to the method described by Remmele et al. [34]. For each specimen, the IRS was calculated as the product of two parameters: staining intensity of the tubular epithelial cytoplasm (graded 0–3; Figure 5A) and the proportion of positively stained area (graded 0–3; Figure 5B), resulting in a final score ranging from 0 to 9.

To minimize potential bias arising from intratumoral heterogeneity in staining patterns within individual specimens, each biopsy sample was subdivided into four distinct quadrants. An individual IRS was calculated for each quadrant, and the sum of the four quadrant-specific scores was used as the final IRS for each specimen (total score range: 0–36; Figure 5C). This standardized approach ensured a more representative and quantitative assessment of the overall protein expression within the renal cortex compartment of the allograft.

### 4.7. Statistical Analysis

All statistical analyses were performed using IBM Statistical Package for the Social Sciences Statistics version 28.0 (IBM Corp., Armonk, NY, USA). Continuous variables were compared between groups using Student’s *t*-test or the Mann–Whitney *U* test, as appropriate, depending on data distribution. Specifically, the Mann–Whitney *U* test was employed for between-group comparisons as well as for longitudinal assessments of the IRS for each mTOR-related protein. Categorical variables were compared using the chi-square test or Fisher’s exact test, as appropriate. In addition, to adjust for potential baseline imbalances and the effect of regression to the mean in IFR at 0 h, ANCOVA was performed, with the 1-year IFR as the outcome variable, baseline (0 h) IFR as the covariate, and the treatment group as the factor. Survival analyses were conducted using the Kaplan–Meier method, and differences between groups were assessed using the log-rank test. For logistic regression analysis, interstitial fibrosis progression was dichotomized based on an increase of 3.8% or more from baseline. Univariate and multivariable logistic regression analyses were then employed to identify independent risk factors for progressive fibrosis formation, and adjusting for both categorical and continuous covariates. A two-sided *p*-value less than 0.05 was considered statistically significant in all analyses.

## 5. Conclusions

Our findings suggest that an EVR-based immunosuppressive regimen attenuates renal interstitial fibrosis from the early post-transplant period by suppressing mTOR-related protein expression without compromising clinical safety. However, long-term survival may be governed by a combination of factors, including specific inflammatory cell infiltrates, rather than by fibrosis alone. Therefore, large-scale prospective studies with long-term follow-up and well-matched cohorts are required to determine whether the antifibrotic effects of EVR ultimately translate into improved long-term allograft survival.

## Figures and Tables

**Table 1:** Clinical Characteristics of Kidney Transplant Recipients and Donors.

	Non-EVR Group (*n* = 43)	EVR Group (*n* = 61)	*p*
Recipients			
Gender (male:female)	28:15	39:22	0.901
Observation period (months)	103.2 ± 43.9	76.9 ± 25.8	<0.001
Age at KTx (years)	48.4 ± 10.3	53.4 ± 12.2	0.014
Dialysis vintage (months)	31.9 ± 33.4	32.5 ± 57.2	0.948
Preemptive KTx, *n* (%)	9 (21)	25 (41)	0.032
Rituximab administration, *n* (%)	11 (26)	23 (38)	0.194
*CYP3A5* (*3/*3:*1/*1 + *1/*3)	31:12	32:29	0.044
PSL withdrawal, *n* (%)	6 (14)	26 (43)	0.002
Primary disease			
CGN, *n* (%)	17 (40)	8 (13)	0.002
DM, *n* (%)	7 (16)	14 (23)	0.404
HT, *n* (%)	1 (2)	9 (15)	0.044
ADPKD, *n* (%)	1 (2)	4 (7)	0.401
Unknown, *n* (%)	17 (40)	26 (43)	0.753
Donors			
Gender (male:female)	14:29	25:36	0.382
Age at KTx (years)	57.4 ± 10.7	60.2 ± 9.0	0.179
Preoperative renal function			
S-Cre (mg/dL)	0.60 ± 0.10	0.68 ± 0.17	0.063
eGFR (mL/min/1.73 m^2^)	86.9 ± 20.5	78.9 ± 18.8	0.047
IFR at 0 h (%)	20.0 ± 3.3	22.5 ± 4.7	0.001

Data are presented as mean ± standard deviation; EVR, everolimus; KTx, kidney transplantation; CYP, cytochrome P450; PSL, prednisolone; CGN, chronic glomerulonephritis; DM, diabetes mellitus; HT, hypertension; ADPKD, autosomal dominant polycystic kidney disease; S-Cre, serum creatinine level; eGFR, estimated glomerular filtration rate; IFR, interstitial fibrosis rate.

**Table 2:** Clinical Outcomes and Pharmacokinetic Parameters of Immunosuppressive Therapy.

	Non-EVR Group (*n* = 43)	EVR Group (*n* = 61)	*p*
Posttransplantation			
S-Cre at 1 month (mg/dL)	1.40 ± 0.60	1.40 ± 0.55	0.481
S-Cre at 1 year (mg/dL)	1.37 ± 0.47	1.40 ± 0.57	0.400
TCMR within 1 year, *n* (%)	20 (47)	19 (31)	0.111
ABMR within 1 year, *n* (%)	4 (9)	2 (3)	0.228
Steroid pulse therapy within 1 year, *n* (%)	14 (33)	14 (23)	0.277
Viral infection			
CMV, *n* (%)	28 (65)	19 (31)	<0.001
BKPyVAN, *n* (%)	0 (0)	3 (5)	0.265
Other viral infections, *n* (%)	4 (9)	7 (12)	0.193
Pharmacokinetics of immunosuppressants			
At 1-month posttransplantation			
TAC trough level (ng/mL)	7.5 ± 1.8	7.7 ± 1.7	0.764
TAC AUC_0–24_ (ng·h/mL)	286.7 ± 67.2	281.0 ± 62.8	0.501
MPA AUC_0–12_ (μg·h/mL)	53.5 ± 21.0	37.4 ± 16.7	<0.001
EVR trough (ng/mL)	-	3.5 ± 1.4	-
EVR AUC_0–12_ (ng·h/mL)	-	52.6 ± 16.6	-
At 1-year posttransplantation			
TAC trough level (ng/mL)	5.5 ± 1.5	5.2 ± 1.3	0.391
TAC AUC_0–24_ (ng·h/mL)	196.0 ± 40.6	175.8 ± 42.1	0.009
MPA AUC_0–12_ (μg·h/mL)	51.5 ± 20.0	39.0 ± 22.4	0.004
EVR trough (ng/mL)	-	4.1 ± 1.5	-
EVR AUC_0–12_ (ng·h/mL)	-	60.8 ± 20.3	-

Data are presented as mean ± standard deviation; EVR, everolimus; S-Cre, serum creatinine level; TCMR, T cell-mediated rejection; ABMR, antibody-mediated rejection; CMV, cytomegalovirus; BKPyVAN, BK polyomavirus-associated nephropathy; TAC, tacrolimus; AUC, area under the blood (plasma) concentration–time curve; MPA, mycophenolic acid.

**Table 3:** Univariate and Multivariable Analyses of Risk Factors Associated with Progression of Interstitial Fibrosis.

	Univariate Analysis			Multivariable Analysis		
	OR	95% CI	*p*	OR	95% CI	*p*
Recipients						
Gender (male vs. female)	0.78	0.34–1.72	0.539			
Age at KTx (<60 vs. 60≤)	1.47	0.62–3.57	0.377			
Dialysis vintage (<15 vs. ≥15 months)	1.47	0.68–3.23	0.327			
Preemptive KTx (yes vs. no)	0.49	0.21–1.13	0.094			
Rituximab administration (yes vs. no)	0.41	0.17–0.96	0.037	0.33	0.09–1.26	0.106
*CYP3A5* genotype (*1/*1 + *1/*3 vs. *3/*3)	0.40	0.18–0.91	0.027	0.61	0.17–2.13	0.438
Primary disease						
CGN (yes vs. no)	1.70	0.68–4.25	0.251			
DM (yes vs. no)	1.13	0.43–2.94	0.807			
HT (yes vs. no)	0.22	0.04–1.09	0.046	0.22	0.02–2.56	0.226
ADPKD (yes vs. no)	0.65	0.11–4.08	1.000			
Posttransplant outcomes						
TCMR within 1 year after KTx (yes vs. no)	1.09	0.49–2.38	0.839			
ABMR within 1 year after KTx (yes vs. no)	1.00	0.19–5.20	1.000			
Steroid pulse therapy within 1 year after KTx (yes vs. no)	1.00	0.42–2.38	1.000			
Viral infection						
CMV (yes vs. no)	1.47	0.68–3.22	0.325			
BKPyVAN (yes vs. no)	0.49	0.04–5.56	1.000			
Others (yes vs. no)	1.87	0.51–6.81	0.339			
EVR administration (yes vs. no)	0.02	0.01–0.07	<0.001	0.03	0.01–0.13	<0.001
PSL withdrawal (yes vs. no)	0.48	0.20–1.14	0.089			
Pharmacokinetics of immunosuppressants at one month after KTx						
TAC trough level (<7.5 vs. ≥7.5 ng/mL)	2.00	0.91–4.35	0.078			
TAC AUC_0–24_ (<280 vs. ≥280 ng·h/mL)	1.18	0.54–2.50	0.695			
MPA AUC_0–12_ (<40 vs. ≥40 ng·h/mL)	0.29	0.13–0.66	0.003	0.40	0.12–1.35	0.143
Pharmacokinetics of immunosuppressants at 1 year after KTx						
TAC trough level (<5 vs. ≥5 ng/mL)	0.95	0.43–2.13	0.900			
TAC AUC_0–24_ (<185 vs. ≥185 ng·h/mL)	0.40	0.18–0.88	0.022	0.71	0.20–2.50	0.589
MPA AUC_0–12_ (<40 vs. ≥40 ng·h/mL)	0.61	0.25–1.41	0.238			
Donors						
Gender (male vs. female)	0.66	0.30–1.47	0.311			
Age at KTx (<60 vs. ≥60)	1.72	0.79–3.70	0.170			
Preoperative renal function						
S-Cre (<0.61 vs. ≥0.61 mg/dL)	1.72	0.78–3.70	0.169			
eGFR (<79.7 vs. ≥79.7 mL/min/1.73 m^2^)	0.68	0.31–1.47	0.327			
IFR at 0 h (<21.2 vs. ≥21.2%)	3.57	1.59–8.00	0.002	1.75	0.50–6.17	0.383

Variables that reached statistical significance (*p* < 0.05) in the univariate analysis were entered into the multivariable analysis. Data are presented as mean ± standard deviation; OR, odds ratio; CI, confidence interval; KTx, kidney transplantation; CYP, cytochrome P450; CGN, chronic glomerulonephritis; DM, diabetes mellitus; HT, hypertension; ADPKD, autosomal dominant polycystic kidney disease; TCMR, T cell-mediated rejection; ABMR, antibody-mediated rejection; CMV, cytomegalovirus; BKPyVAN, BK polyomavirus-associated nephropathy; EVR, everolimus; PSL, prednisolone; TAC, tacrolimus; AUC, area under the blood concentration–time curve; MPA, mycophenolic acid; S-Cre, serum creatinine level; eGFR, estimated glomerular filtration rate; IFR, interstitial fibrosis rate.

## Data Availability

The original contributions presented in this study are included in the article. Further inquiries can be directed to the corresponding author.

## Abbreviations

The following abbreviations are used in this manuscript:

4EBP1	Eukaryotic translation initiation factor 4E-binding protein 1
ABMR	Antibody-mediated rejection
ADPKD	Autosomal dominant polycystic kidney disease
AUC	Area under the concentration–time curve
BKPyVAN	BK polyomavirus-associated nephropathy
CGN	Chronic glomerulonephritis
CI	Confidence Interval
CMV	Cytomegalovirus
CNI	Calcineurin inhibitor
CYP	Cytochrome P450
DM	Diabetes mellitus
DSA	Donor-specific antibody
eGFR	Estimated glomerular filtration rate
EM	Elastica–Masson
EVR	Everolimus
HR	Hazard Ratio
HT	Hypertension
IFR	Interstitial fibrosis rate
IRS	Immunoreactive score
KTx	Kidney transplantation
MMF	Mycophenolate mofetil
MPA	Mycophenolic acid
mTOR	Mammalian target of rapamycin
mTORi	Mammalian target of rapamycin inhibitor
N.S.	Not significant
p-4EBP1	Phosphorylated eukaryotic translation initiation factor 4E-binding protein 1
p-P70S6K	Phosphorylated p70 ribosomal S6 kinase
PSL	Prednisolone
RGB	Red, green, and blue
S-Cre	Serum creatinine level
TAC	Tacrolimus
TCMR	T cell-mediated rejection
TGF-β	Transforming growth factor-beta
